# Association between the geriatric nutritional risk index and cognitive functions in older adults: a cross-sectional study from National Health and Nutrition Examination Survey

**DOI:** 10.3389/fnut.2024.1459638

**Published:** 2024-08-14

**Authors:** Zhichun Tan, Yaxin Nie, Ning Yan

**Affiliations:** Neurology Department, The Affiliate University-Town Hospital, Chongqing Medical University, Chongqing, China

**Keywords:** cross-sectional study, geriatric nutritional risk index, older adults, cognitive function, nutritional status, NHANES

## Abstract

**Objective:**

To investigate the associations between the geriatric nutritional risk index (GNRI) with cognitive functions among U.S. older adults. (Patients were classified into two nutrition risk groups based on the GNRI).

**Methods:**

Our analysis utilized data from the cross-sectional National Health and Nutrition Examination Survey (NHANES) conducted between 2011 and 2014. Cognitive function was measured using CERAD test, AFT and DSST. Composite *z*-scores were obtained by summing test-specific *z*-scores of the above three cognitive tests and were used to assess the global cognitive function. We employed weighted logistic regression models to evaluate the associations between GNRI and nutritional status (low and high GNRI) with cognitive function among older participants. The non-linear relationship was described using fitted smoothed curves and threshold effect analyses. Subgroup analysis and interaction tests were also conducted.

**Results:**

This study included 2,592 older participants aged 60 years and older. After adjusting for confounding variables, the GNRI was positively associated with AFT (*β* = 0.05, 95% CI 0.005–0.096, *p*-value = 0.0285), DSST (*β* = 0.192, 95% CI 0.078–0.305, *p*-value = 0.0010) and the composite *z*-scores (*β* = 0.027, 95% CI 0.010–0.044, *p*-value = 0.0024). The results also showed that the high-GNRI group was significantly associated with AFT (*β* = 0.922, 95% CI 0.166–1.677, *p*-value = 0.0169), DSST (*β* = 2.791, 95% CI 0.884–4.698, *p*-value = 0.0042) and composite *z*-scores (*β* = 0.405, 95% CI 0.115–0.695, *p*-value = 0.0062) likewise had significant positive correlations, using the low-GNRI group as a reference. In addition, inflection points with CERAD and composite *z*-scores were found at GNRI of 108.016, and 105.371, respectively. Specifically, on the left side of the inflection point GNRI levels were positively correlated with CERAD and composite *z*-scores (CERAD *β* = 0.087, 95% CI 0.024–0.150, *p*-value = 0.0070; composite *z*-scores *β* = 0.065, 95% CI 0.040–0.091, *p*-value <0.0001), while on the right side of the inflection point were significantly negatively associated (CERAD *β* = −0.295, 95% CI −0.529 to −0.062, *p*-value = 0.0133, composite *z*-scores *β* = −0.050, 95% CI −0.091 to −0.008, *p*-value = 0.0184).

**Conclusion:**

Lower GNRI was associated with poorer performance in several cognitive domains. Additionally, there was a non-linear positive association between GNRI and cognitive function in normal nutritional states, for excessive GNRI may cause cognitive decline.

## 1 Introduction

The increasing incidence of cognitive function deterioration is becoming a significant concern in the context of global aging (1). Cognitive decline refers to the worsening of abilities across various cognitive domains, such as memory, language, judgment, executive functions, visuospatial skills and computational capabilities (2). Without timely prevention and intervention, this decline can evolve into mild cognitive impairment (MCI) and ultimately progress irreversibly to different forms of dementia, predominantly Alzheimer’s disease (3, 4). This progression poses substantial challenges to the quality of life of not only the affected individuals but also their families and society at large (5). Once dementia is established, the outcomes from existing treatments are frequently unsatisfactory (6). However, preemptive measures including lifestyle modifications like enhanced nutrition and increased physical activity can effectively manage and potentially prevent the deterioration of cognitive functions (7).

Previous studies have shown that diet and nutrition, as a common lifestyle, are significantly associated with CI and dementia (8–10). Two studies have relied on specific nutritional markers like vitamin and albumin levels (11, 12) or utilized scales such as the mini nutritional assessment (MNA) and its short form (MNA-SF) to investigate the link between malnutrition and cognitive function (13, 14). However, some of the above criteria for assessing nutritional status are subjective and do not fully reflect the overall nutritional status, so the relationship between nutritional status and cognitive function is still controversial. The geriatric nutritional risk index (GNRI), which is a more straightforward dietary index that evaluates the nutritional status of older adults using objective criteria such as height, weight, ideal body weight, and albumin levels (15), has recently been acknowledged as equally effective as the MNA in determining malnutrition (16).

To date, research examining the link between the GNRI and normal nutritional status with cognitive function remains scarce. In this investigation, we evaluated the relationship between GNRI and cognitive performance using a representative cohort of older U.S. adults from the 2011–2014 National Health and Nutrition Examination Survey (NHANES). Additionally, we analyzed how GNRI levels correlate with cognitive domains, through the application of three tests among older adults who are in a normal nutritional state.

## 2 Methods

### 2.1 Study population

The cohort for our study was sourced from the NHANES, which assesses the health and nutritional status of a representative segment of the U.S. populace through complex and multistage stratified random sampling. NHANES data has been collected continuously since 1999, and data are released publicly in 2-year cycles. The study’s protocol was sanctioned by the National Center for Health Statistics (NCHS), and all participants provided informed consent. We can find NHANES database from the link: https://www.cdc.gov/nchs/nhanes/index.htm.

**Figure 1:**
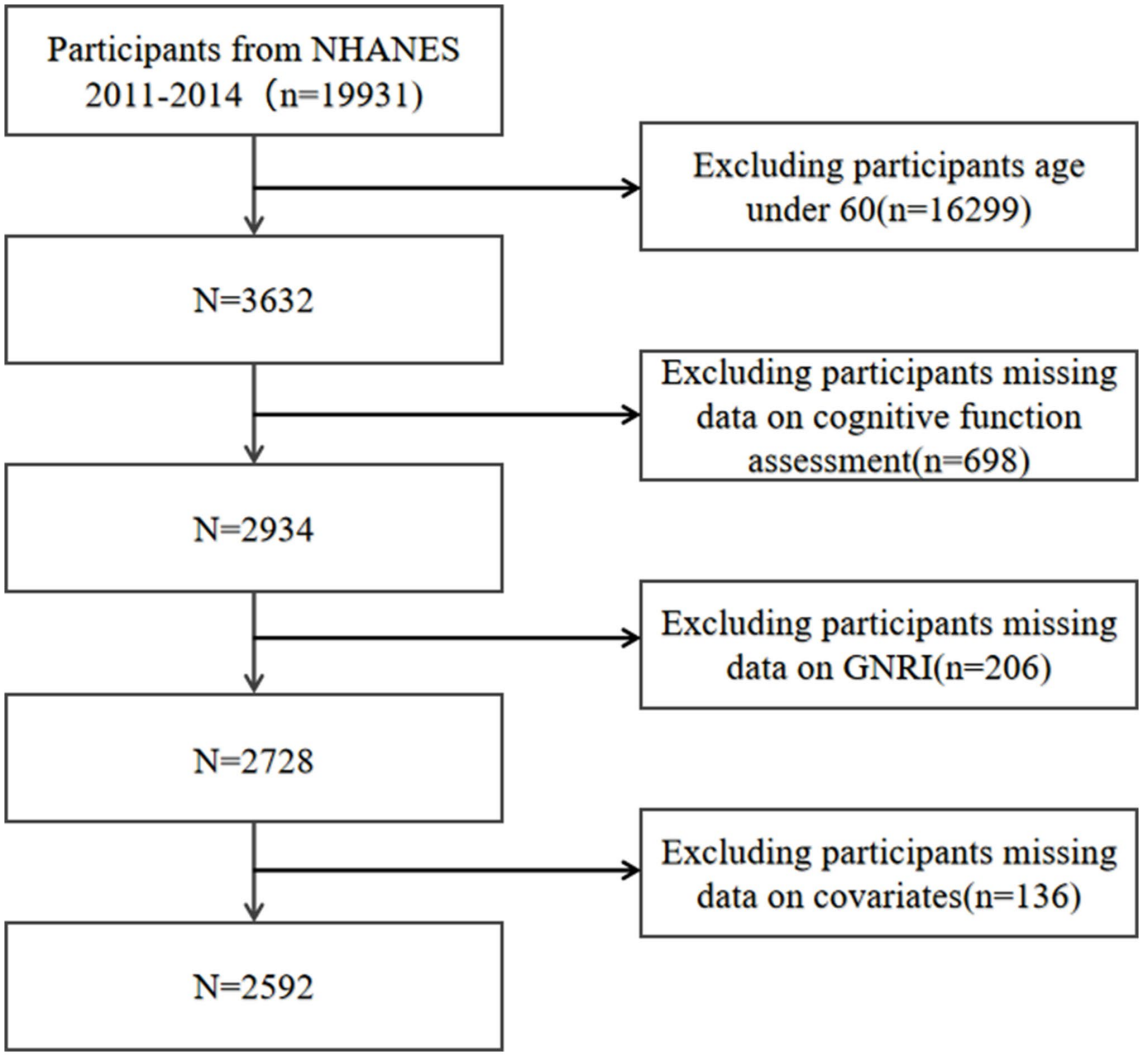
Flow chart of participants selection. NHANES, National Health and Nutrition Examination Survey.

Our analysis incorporated individuals from the 2011 to 2014 NHANES cycles, as these were the periods during which the latest assessments of cognitive function were available. Initially, we included 19,931 participants. Exclusions were made for those under the age of 60, resulting in 16,299 participants being omitted. Additionally, participants missing cognitive function test data (*n* = 698), GNRI-related data (*n* = 206), and necessary covariates (*n* = 136) were also excluded. Eventually, 2,592 participants remained eligible for inclusion in our research (Figure 1).

### 2.2 GNRI assessment

GNRI is a newly nutritional assessment tool designed to evaluate the nutritional risk and its associated morbidity and mortality in older adults. According to previous studies (17, 18), the GNRI is composed of the individual’s height (m), weight (m), ideal weight (kg), and serum albumin (g/L). The calculation formula as follows (18): GNRI = 1.489 × albumin (g/L) + 41.7 × (weight/ideal weight), ideal weight = 22 × height (m) × height (m). In cases where the actual weight exceeded the ideal weight, the set weight/ideal weight was 1. In our study, the total GNRI was treated as a continuous variable and participants were divided into two categories based on their GNRI levels (18): a low-GNRI group (GNRI <98) and a high-GNRI group (GNRI ≥98). The low-GNRI group included individuals identified as malnourished, whereas the high-GNRI group comprised those with normal nutritional status. The GNRI was designated as the exposure variable for the research.

### 2.3 Cognitive function assessment

To assess the cognitive performance in participants, we employed the following three cognitive function tests, with higher scores indicative of better cognitive function.

The consortium for the establishment of an Alzheimer’s Disease Registry (CERAD) test involves three consecutive learning trials and one delayed recall trial, aimed at evaluating the immediate and delayed acquisition of new verbal information (19), with scores from 1 to 40. The animal fluency test (AFT) assesses categorical language fluency (20), with scores ranging from 3 to 40. The digit symbol substitution test (DSST), a component of the Wechsler Adult Intelligence Scale, measures processing speed, sustained attention, and working memory (21), with scores between 0 and 105.

Additionally, we calculated test-specific *z*-scores for each cognitive test by using the sample mean and standard deviation of the scores from each of the three listed cognitive tests, based on the prior reference [22]. These specific *z*-scores were then aggregated to create a composite *z*-score. The composite *z*-score was utilized to assess the global cognitive function of the study participants.

### 2.4 Covariates

We included relevant covariates that could potentially influence the association between GNRI and cognitive function in three categories: sociodemographic variables, lifestyle, and common diseases in older adults (23, 24).

Sociodemographic variables included sex (male/female), age (years), race (Mexican American, other Hispanic, non-Hispanic White, non-Hispanic Black, other), poverty-to-income ratio (PIR), education levels (less than high school, high school and above), and body mass index (BMI, kg/m^2^). Lifestyle factors comprised physical activity (active/inactive), smoking status (yes/no) and drinking status (yes/no). Common diseases in older adults that were considered included diabetes, hypertension, self-reported cardiovascular diseases (CVD) and stroke. Physically active was defined as engaging in at least 10 consecutive minutes of moderate or vigorous intensity activities not related to work or transport. While activities lasting less than 10 min were classified as physically inactive (25). Smoking status was assessed based on whether an individual had smoked at least 100 cigarettes in their lifetime. Drinking status was identified by whether an individual had at least 12 drinks per year. Self-reported CVD included congestive heart failure, coronary heart disease, angina/angina pectoris, and heart attack. Hypertension was determined either through a mean systolic blood pressure exceeding 140 mmHg or a mean diastolic blood pressure over 90 mmHg from the last three measurements, or by self-reported history. Diabetes was diagnosed either through physician diagnosis or the glycosylated hemoglobin level exceeding 6.5%.

### 2.5 Statistical analysis

In the weighted baseline table, continuous variables were described using the mean ± standard deviation, while categorical variables were represented as percentages. To explore differences across subgroups defined by varying GNRI levels, we applied *t*-tests for continuous variables and chi-squared tests for categorical variables. We also conducted analyses using weighted multivariate logistic regression to compute beta values and 95% confidence intervals, aiming to assess the association between GNRI and cognitive function. In our analytical models, no covariates were adjusted in model 1. Model 2 included adjustments for gender, age, and race. Model 3 accounted for all covariates. Furthermore, we performed smoothed curve fitting and threshold effects analyses to further investigate the non-linear relationship between GNRI and cognitive function, including identifying potential inflection points, adjusting for all covariates. Finally, to assess the stability of the correlation between high-GNRI and global cognitive function, we conducted further subgroup analyses and interaction tests, adjusting for all confounding factors in the process. Missing values for continuous variables in this study were entered from the median or mean of available cases for these variables. All study analyses were completed in EmpowerStats (versions 2.0; http://www.empowerstats.com) and R software (version 4.2.2; http://www.r-project.org).

## 3 Results

### 3.1 Baseline characteristics of participants

The baseline characteristics of the 2,592 participants in our study, stratified by GNRI levels, are summarized in Table 1. The weighted average age of the study was 69.08 ± 6.63 years, with a slightly higher proportion of females (53.69%) compared to males (46.31%). Compared with the low-GNRI group, the high-GNRI group was younger and more likely to be male, had a higher proportion of other Hispanics and non-Hispanic White people; was more likely to be overweight (BMI ≥25); had higher alcohol consumption and physical activity status; and had a lower prevalence of stroke and diabetes. In addition, the high-GNRI group scored higher on all three cognitive function tests and had higher composite *z*-scores compared to the low-GNRI group.

**Table 1:** Weighted characteristics of study population based on different GNRI levels.

Variables	Low-GNRI (*N* = 225)	High-GNRI (*N* = 2,367)	*p*-value
Age (years)	70.86 ± 7.09	68.94 ± 6.57	0.0001
**Gender (%)**			<0.0001
Male	32.18	47.40	
Female	67.82	52.60	
**Race (%)**			0.0073
Mexican American	3.99	3.21	
Other Hispanic	2.88	3.63	
Non-Hispanic White	73.32	81.10	
Non-Hispanic Black	14.59	7.24	
Other race	5.22	4.82	
**Education level (%)**			0.2198
<High school	18.76	15.37	
High school, or >high school	81.24	84.63	
**PIR (%)**			0.1137
≤1	11.29	7.98	
>1	88.71	92.02	
**BMI (%)**			0.0024
<25	36.29	25.44	
≥25, <30	27.69	37.15	
≥30	36.02	37.41	
**Smoking status (%)**			0.6125
Yes	48.31	50.24	
No	51.69	49.76	
**Drinking status (%)**			0.0033
Yes	64.15	74.05	
No	35.85	25.95	
**Physical activity (%)**			<0.0001
Active	31.24	46.86	
Inactive	68.76	53.14	
**CVD (%)**			0.2942
Yes	20.15	17.13	
No	79.85	82.87	
**Stroke (%)**			0.0002
Yes	12.37	5.53	
No	87.63	94.47	
**Hypertension (%)**			0.5630
Yes	64.58	66.66	
No	35.42	33.34	
**Diabetes (%)**			0.0326
Yes	31.87	24.80	
No	68.13	75.20	
Composite *z*-score	−0.10 ± 2.35	0.84 ± 2.41	<0.0001
CERAD test score	25.04 ± 6.84	26.13 ± 6.30	0.0243
AFT score	16.20 ± 4.83	18.30 ± 5.64	<0.0001
DSST score	46.36 ± 17.43	53.11 ± 16.37	<0.0001

Mean ± SD for continuous variables: *p*-value was calculated by weighted linear regression model. % for categorical variables: *p*-value was calculated by weighted chi-square test.PIR, poverty-income ratio; BMI, body mass index; CVD, cardiovascular disease; CERAD, the consortium to establish a registry for Alzheimer’s disease; AFT, animal fluency test; DSST, digit symbol substitution test.The composite *z*-score was calculated by summing the *z*-scores [(test score − mean score)/SD] of the three individual tests.

### 3.2 Composite *z*-scores distribution of participants in the high-GNRI and low-GNRI group

**Figure 2:**
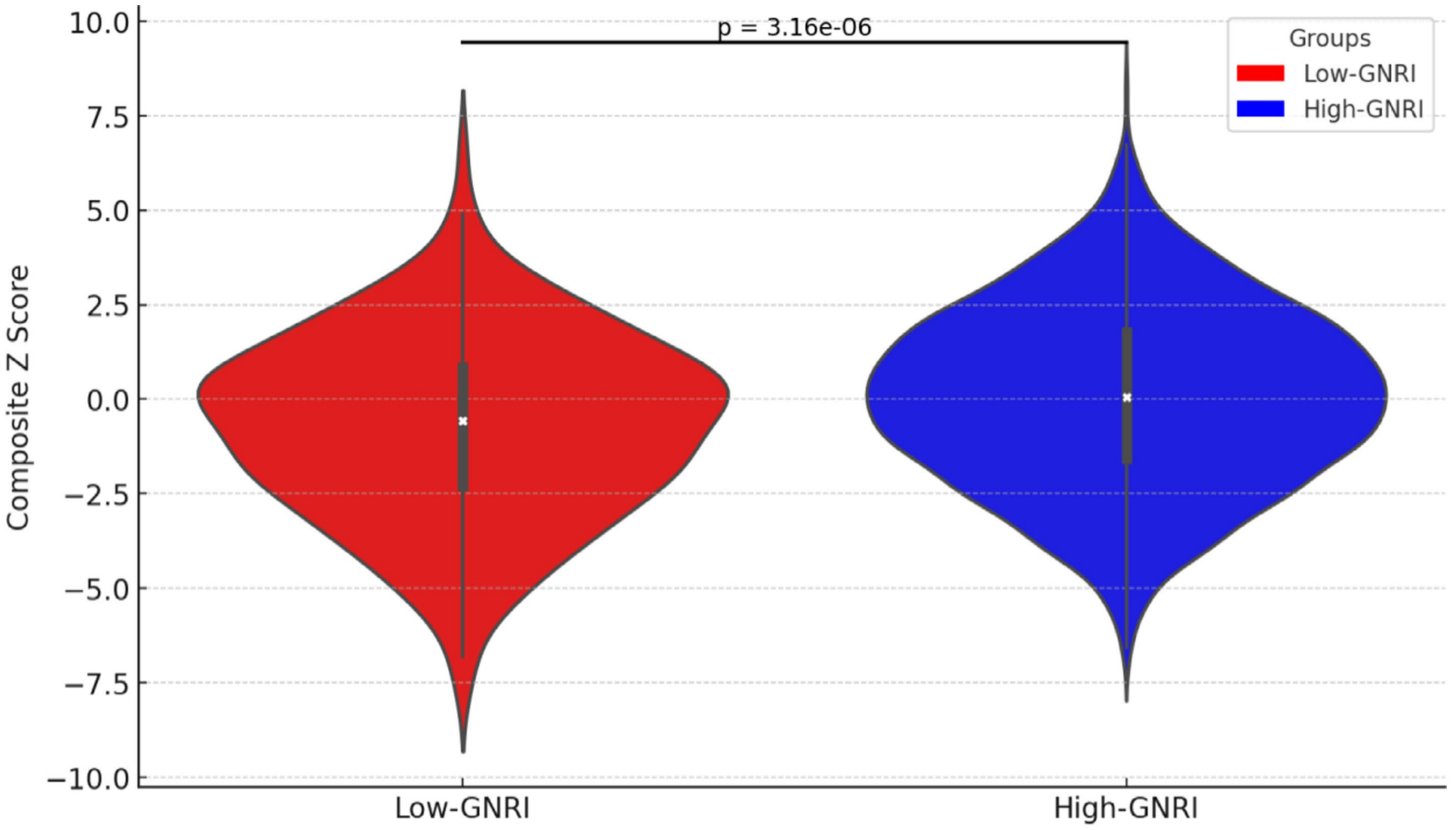
Violin plot of composite *z*-scores distribution in high-GNRI and low-GNRI group participants.

Participants in the high-GNRI group had higher composite *z*-scores compared to the low-GNRI group, suggesting higher global cognitive function in the high-GNRI group population. And the difference was statistically significant (*p*-value <0.0001) (Figure 2).

### 3.3 The association between GNRI levels and cognitive function

The outcomes of the multivariate logistic regression analyses are detailed in Table 2. After adjusting for all covariates, the significant positive correlation was observed between total GNRI and scores from the AFT, DSST, and composite *z*-scores. Specifically, for each unit increase in total GNRI, there was an increase of 0.050 points in AFT scores (*β* = 0.050, 95% CI 0.005–0.096, *p*-value = 0.0258), 0.192 points in DSST scores (*β* = 0.192, 95% CI 0.078–0.305, *p*-value = 0.0010), and 0.027 points in composite *z*-scores (*β* = 0.027, 95% CI 0.010–0.044, *p*-value = 0.0024). Additionally, when analyzed by GNRI levels, the GNRI levels in the high-GNRI group were significantly associated with better outcomes in cognitive functioning. Compared to the low-GNRI group, the high-GNRI group exhibited significantly higher AFT scores (*β* = 0.922, 95% CI 0.166–1.677, *p*-value = 0.0169) and DSST scores (*β* = 2.791, 95% CI 0.884–4.698, *p*-value = 0.0042). Furthermore, the higher GNRI levels were also significantly and positively correlated with higher composite *z*-scores, reflecting overall cognitive functioning (*β* = 0.405, 95% CI 0.115–0.695, *p*-value = 0.0062).

**Table 2:** Logistic regression analysis for associations between GNRI and cognitive function scores.

Cognitive function	Model 1 [*β* (95% CI)]	*p*-value	Model 2 [*β* (95% CI)]	*p*-value	Model 3 [*β* (95% CI)]	*p*-value
**CERAD**						
Total GNRI	0.089 (0.033, 0.144)	0.0018	0.061 (0.009, 0.112)	0.0222	0.041 (−0.011, 0.093)	0.1185
Low-GNRI	Reference		Reference			Reference
High-GNRI	1.089 (0.142, 2.035)	0.0243	0.654 (−0.223, 1.532)	0.1441	0.474 (−0.396, 1.343)	0.2860
**AFT**						
Total GNRI	0.014 (0.091, 0.189)	<0.0001	0.083 (0.037, 0.129)	0.0004	0.050 (0.005, 0.096)	0.0285
Low-GNRI	Reference		Reference		Reference	
High-GNRI	2.097 (1.262, 2.931)	<0.0001	1.252 (0.473, 2.032)	0.0017	0.922 (0.166, 1.677)	0.0169
**DSST**						
Total GNRI	0.495 (0.351, 0.638)	<0.0001	0.339 (0.217, 0.461)	<0.0001	0.192 (0.078, 0.305)	0.0010
Low-GNRI	Reference		Reference		Reference	
High-GNRI	6.749 (4.292, 9.205)	<0.0001	4.343 (2.270, 6.416)	<0.0001	2.791 (0.884, 4.698)	0.0042
**Composite *z*-score**						
Total GNRI	0.068 (0.047, 0.089)	<0.0001	0.044 (0.026, 0.062)	<0.0001	0.027 (0.010, 0.044)	0.0024
Low-GNRI	Reference		Reference		Reference	
High-GNRI	0.946 (0.588, 1.305)	<0.0001	0.584 (0.275, 0.893)	<0.0001	0.405 (0.115, 0.695)	0.0062

Model 1: no adjustment for covariates.Model 2: age, gender, and race adjusted.Model 3: age, race, gender, BMI, education level, PIR, physical activity, smoking and drinking status, CVD, stroke, diabetes, hypertension. CERAD, the consortium to establish a registry for Alzheimer’s disease; AFT, animal fluency test; DSST, digit symbol substitution test.The composite *z*-score was calculated by summing the *z*-scores [(test score − mean score)/SD] of the three individual tests.

### 3.4 Non-linear relationship between the GNRI and cognitive performance

**Figure 3:**
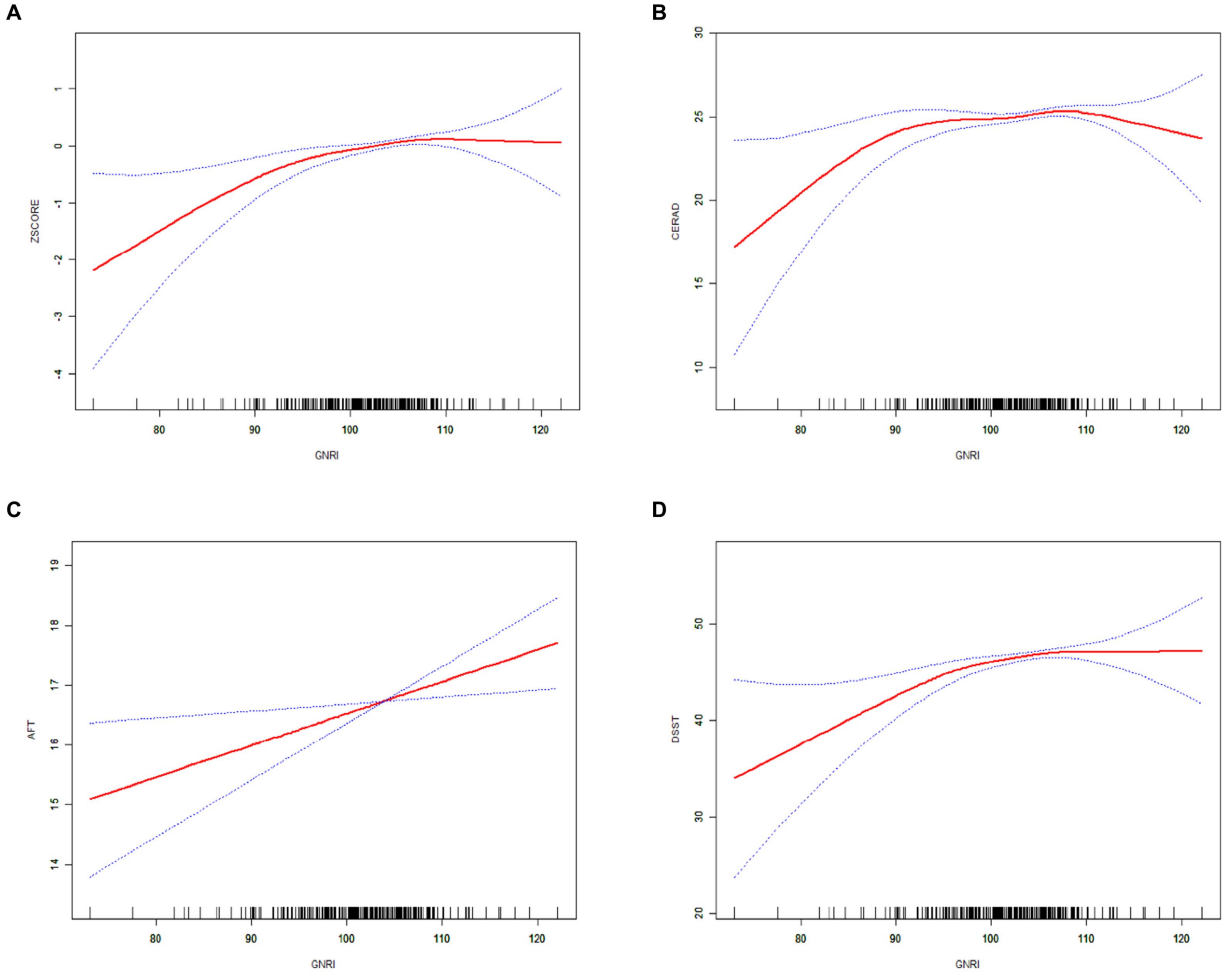
The solid red line indicates a smooth curve fit between the GNRI and cognitive performance. Blue bars indicate 95% of the fitted confidence intervals. **(A)** GNRI and composite *z*-score. **(B)** GNRI and CERAD. **(C)** GNRI and AFT. **(D)** GNRI and DSST.

The non-linear positive association between the GNRI and the three cognitive test scores, as well as the composite *z*-scores, were confirmed through smoothed curve fitting analyses, as shown in Figure 3. Furthermore, the non-linear U-shaped relationship between GNRI and global cognitive function was identified using a two-segment linear regression model, with calculated threshold effects presented in Table 3.

**Table 3:** Threshold effect analysis of GNRI on cognitive function using a two-segment linear regression model.

Outcomes	CERAD *β* (95% CI) *p*-value	AFT *β* (95% CI) *p*-value	DSST *β* (95% CI) *p*-value	Composite *z*-score *β* (95% CI) *p*-value
**Linear effect model**				
	0.041 (−0.011, 0.093) 0.1185	0.050 (0.005, 0.095) 0.0285	0.192 (0.078, 0.305) 0.0010	0.027 (0.010, 0.044) 0.0024
**Non-linear model**				
Inflection point (K)	108.016	104.219	96.793	105.371
GNRI <K	0.087 (0.024, 0.150) 0.0070	0.156 (0.082, 0.230) <0.0001	1.119 (0.643, 1.595) <0.0001	0.065 (0.040, 0.091) <0.0001
GNRI >K	−0.295 (−0.529, −0.062) 0.0133	−0.089 (−0.178, 0.001) 0.0514	0.062 (−0.069, 0.192) 0.3549	−0.050 (−0.091, −0.008) 0.0184
Log likelihood ratio	0.013	<0.001	<0.001	<0.001

Age, race, gender, BMI, education level, PIR, physical activity, smoking and drinking status, CVD, stroke, diabetes, and hypertension were adjusted. CERAD, the consortium to establish a registry for Alzheimer’s disease; AFT, animal fluency test; DSST, digit symbol substitution test.The composite *z*-score was calculated by summing the *z*-scores [(test score − mean score)/SD] of the three individual tests.

The smoothed curve depicting the relationship between GNRI and the global cognitive function revealed an inflection point at a GNRI value of 105.37. On the left side of this inflection point, there was a positive association with an increase in GNRI associated with improved cognitive scores (CERAD *β* = 0.087; 95% CI: 0.024, 0.150; *p*-value = 0.0070; composite *z*-scores *β* = 0.065; 95% CI: 0.040, 0.091; *p*-value <0.0001). Conversely, on the right side of the inflection point, the association turned negative, indicating that higher GNRI values beyond this point were associated with cognitive decline (CERAD *β* = −0.295; 95% CI: −0.529, −0.062; *p*-value = 0.0133, composite *z*-scores *β* = −0.050; 95% CI: −0.091, −0.008; *p*-value = 0.0184).

### 3.5 Subgroup analysis

**Figure 4:**
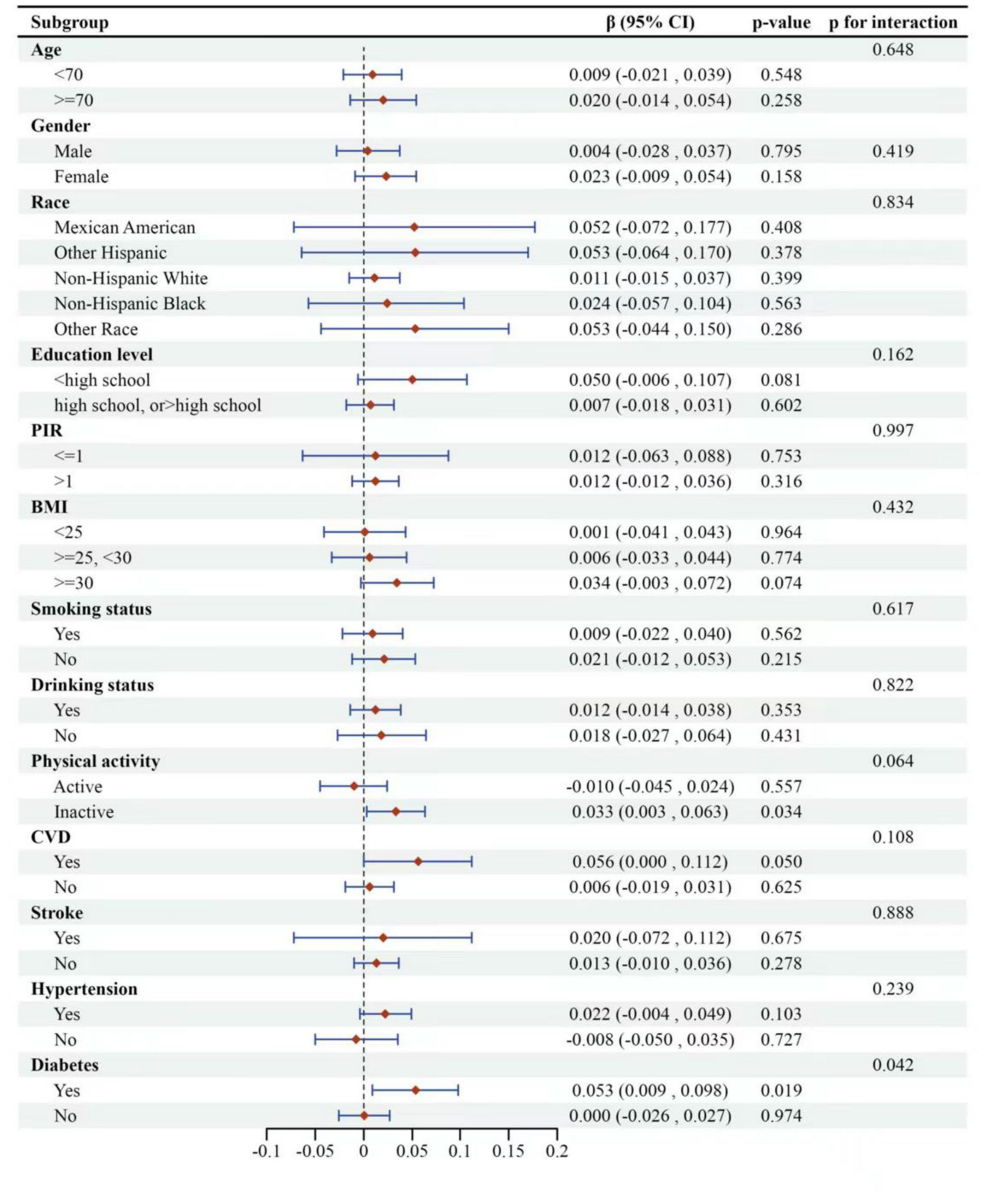
Subgroup logistic regression analysis for the association between high-GNRI and global cognitive function. The global cognitive function was represented by composite *z*-scores. Low-GNRI was the control group.

Finally, we implemented adaptive subgroup analyses and interaction tests stratified by all confounders to evaluate the consistency of the correlation between high GNRI and overall cognitive functioning, and to identify potential differences among specific populations, as depicted in Figure 4. The results indicated that participants with diabetes exhibited a significant positive correlation between overall cognitive function and GNRI. This positive correlation was notably absent in non-diabetic patients, with the interaction effect reaching statistical significance (*p* for interaction = 0.0423). Furthermore, participants with a history of cardiovascular disease (CVD) demonstrated a tendency to have higher cognitive functioning with increased GNRI, which was marginally significant (*p*-value = 0.0498).

Among the other covariates examined, the association between GNRI and global cognitive function remained consistent across the remaining subgroups, with no significant interactions observed (*p* for interaction >0.05). The analysis suggested that GNRI’s impact on cognitive function may vary depending on the presence of specific health conditions like diabetes and CVD.

## 4 Discussion

Our results demonstrated that the GNRI is significantly and positively correlated with the AFT, DSST and composite *z*-scores, even after adjusting for potential confounding variables. These findings indicated that a lower GNRI is linked to declines across multiple cognitive domains and global cognitive deterioration.

Upon categorizing participants based on their nutritional status (GNRI <98; GNRI ≥98), our analysis revealed a positive correlation between GNRI and cognitive functioning in older adults who maintain a normal nutritional status (GNRI ≥98), as opposed to those considered malnourished (GNRI <98).

Previous investigations into the association between the GNRI and cognitive function in older adults have been limited, with most research focusing on malnutrition as assessed by GNRI and yielding mixed results. For instance, a longitudinal analysis from the Chinese Longitudinal Healthy Longevity Survey (CLHLS) found a linear relationship between malnutrition risk (GNRI ≤98) and declining cognitive function, indicating that worsening malnutrition correlates with deteriorating cognitive abilities (26). Additionally, a study of an elderly Chinese stroke population identified a low GNRI (<98) as a predictive marker for post-stroke cognitive impairment (27). However, other studies have reported differing outcomes; for example, Lee et al. (28) found no significant association between GNRI and cognitive impairment risk among hemodialysis patients. And Xu et al. (29) observed no significant link between GNRI and cognitive function indicators in elderly patients with coronary artery disease. These divergent findings may be attributable to the specific diseases affecting the study populations or the varying methodologies employed in assessing cognitive function. Consistent with some prior studies, our research also observed lower cognitive function among malnourished participants, highlighting the ongoing need for extensive research to further elucidate the relationship between GNRI and cognitive function.

Additionally, previous evidence suggested that cognitive decline and the progression of dementia can potentially be mitigated through nutritional supplementation and maintaining a healthy diet (30). Notably, the combined intake of multiple B vitamins such as folic acid, vitamin B6, and vitamin B12 has been shown to prevent cognitive decline by inhibiting the accumulation of homocysteine (31–33). Furthermore, there is an expanding body of evidence supporting the role of *n* − 3 fatty acids, vitamins D and E, and plant-derived vitamins in preventing cognitive deterioration and potentially dementia (33–36). On the other hand, an increasing number of studies have identified a negative correlation between the consumption of trans fats and added sugars and cognitive function (37, 38), suggesting that higher nutritional status may paradoxically lead to diminished cognitive performance. This aligns with our findings where smoothed curve fitting and threshold effect analyses revealed that higher GNRI levels were negatively associated with both CERAD scores and composite *z*-scores as GNRI increased. Consequently, a higher nutritional status does not necessarily eliminate the risk of cognitive decline. Effective prevention might be achievable through maintaining an optimal GNRI by adopting a balanced nutritional intake and following a healthy dietary pattern (39).

The mechanism by which the GNRI appears to be associated with cognitive function is unclear. The main reason may be that GNRI is a valid indicator for assessing nutritional status, which has been shown to be strongly associated with cognitive function (16–18), although the exact mechanism is still unclear. GNRI is calculated on the basis of height (cm), body weight (kg), ideal body weight (kg), and serum albumin (18). Therefore, the relationship between GNRI and cognitive function may be related to albumin and BMI. Recent studies have shown that higher albumin levels are associated with a lower risk of cognitive impairment, suggesting that maintaining high levels of albumin may benefit cognitive function in older adults (12, 40, 41). This may be because albumin itself is closely related to nutritional status (42). Reduced albumin levels may interfere with the blood supply to the central nervous system and may disrupt the oxidant/antioxidant balance, contributing to the development of cognitive impairment (43–46). Similarly, BMI, an assessment index for obesity, has been consistently shown to be strongly associated with cognitive function, but the findings were controversial. Some studies have suggested that overweight older adults have a lower risk of developing CI or dementia (47, 48). However, recent studies have shown that higher BMI is associated with poorer cognitive functioning in older adults (49, 50), which may also account for the cognitive decline seen in participants with excessive GNRI. And some studies have found significant correlations between GNRI and the prevalence of diabetes, stroke, and depression (18, 51, 52). Therefore, it is possible that GNRI indirectly affects cognitive function through the above factors (53–55).

The study addressed the underexplored relationship between the geriatric nutritional risk index (GNRI) and cognitive function among a representative cohort of U.S. older adults. We found a significant positive correlation between GNRI and cognitive function. However, excessively high GNRI values were also associated with declines in cognitive performance. This suggested that GNRI could potentially serve as a reliable predictor of cognitive decline in clinical settings, further emphasizing the link between malnutrition and cognitive function.

Our research, a cross-sectional analysis based on data from the NHANES, benefited from the representativeness of the NHANES sample, providing a more accurate reflection of the older American population compared to other studies. We utilized various cognitive tests to evaluate different cognitive abilities among older adults, alongside composite *z*-scores for an overall assessment of cognitive function. The substantial sample size facilitated subgroup analyses, enhancing our understanding of the GNRI-cognitive function relationship across different demographics. Nonetheless, our study is not without limitations. The cross-sectional nature of NHANES restricts our ability to track changes in nutritional status and cognitive function over time or to establish a causal relationship between these variables. Additionally, some variables in NHANES come from questionnaires and self-reports, which are prone to biases. And the evidence from the U.S. may not be generalizable to other populations due to differences in genetic origin and socioeconomics conditions. Furthermore, of a total 3,632 elderly people, 1,040 (28%) were excluded due to missing data on covariates, which may have affected the results. Consequently, future multicenter longitudinal clinical trials with extended follow-ups are essential to more definitively determine the relationship between GNRI and cognitive function in the aging population.

## 5 Conclusion

In a representative cohort of older Americans, our study reinforced the link between malnutrition and cognitive decline. Additionally, our findings indicated that higher geriatric nutritional risk index (GNRI) levels correlate with improved cognitive function. However, there may be instances where increased GNRI levels within a normal nutritional status could lead to cognitive deterioration. This implies that GNRI could serve as a valuable tool for predicting changes in cognitive function in clinical environments in the future.

## Data Availability

The original contributions presented in the study are included in the article/supplementary material, further inquiries can be directed to the corresponding author.
